# Role of microbes in colorectal cancer therapy: Cross-talk between the microbiome and tumor microenvironment

**DOI:** 10.3389/fphar.2022.1051330

**Published:** 2022-11-08

**Authors:** Cong Xia, Yantao Cai, Shuangyi Ren, Chenglai Xia

**Affiliations:** ^1^ Department of Gastrointestinal Surgery, The Second Affiliated Hospital of Dalian Medical University, Dalian, China; ^2^ Affiliated Foshan Maternity and Child Healthcare Hospital, Southern Medical University, Foshan, China; ^3^ School of Pharmaceutical Sciences, Southern Medical University, Guangzhou, China

**Keywords:** tumor microenvironment, immunocyte, microbioma, cytokin, colorectal cancer

## Abstract

The human gut microbiota is associated with the development and progression of colorectal cancer, and manipulation of the gut microbiota is a novel strategy for the prevention and treatment of colorectal cancer. Some bacteria have antitumor activity against colorectal cancer, where specific bacteria can improve the tumor microenvironment, activate immune cells including dendritic cells, helper T cells, natural killer cells, and cytotoxic T cells, and upregulate the secretion of pro-tumor immune cytokines such as interleukin-2 and interferon. In this paper, we summarize some bacteria with potential benefits in colorectal cancer and describe their roles in the tumor microenvironment, demonstrate the application of gut microbes in combination with immunosuppressive agents, and provide suggestions for further experimental studies and clinical practice applications.

## Introduction

The occurrence and development of colorectal cancer (CRC) are closely related to the intestinal flora, with some microorganisms exerting a tumor-promoting effect and others exerting an inhibitory effect. Intestinal microbes colonize near tumors and participate in constituting and influencing the tumor microenvironment (TME). Some beneficial gut microbes can influence host immunity and affect immune cells and cytokines in the TME, thus exerting anti-tumor immune effects. With the application and development of tumor immunotherapy, the role of gut microbiota in tumor immunity has been emerging. We have studied some beneficial microorganisms and outlined their essential roles in tumor immunity.

## The relationship between microorganisms and CRC

The human microbiota colonizes the gastrointestinal tract, oropharyngeal cavity, skin, and genitourinary urogenital tract. There are 3.8×10^13^ bacteria in the human body, more than the number of cells in the human body (Sender, et al., 2016). The gut microbiota is composed of different species of bacteria, archaea, fungi, protozoa, and viruses (El-Sayed, et al., 2021). The colon is a favorable environment for bacterial growth because of its high pH value, low antimicrobial content, and low bile acid concentration (Donaldson, et al., 2016). At the same time, the oxygen content in the colon is low; intestinal bacteria are mainly anaerobic. Most of the bacteria are *Bacteroidetes* and *Firmicutes*. There are also a few other bacteria, such as *Actinobacteria* and *Proteobacteria* (Kwon, et al., 2021). The gut microbiota is involved in host physiological processes, such as nutrition, metabolism, and immunity. Dysbiosis of the gut microbiota is associated with CRC (Kong and Cai, 2019). In CRC, the microbiome generally has increased bacteria such as *Bacteroides*, *Parabacteroides*, *Alistipes*, *Akkermansia spp.*, Porphyromonadaceae, Corynebacteriaceae, Staphylococcaceae, and *Methanobacteriales*, whereas other bacteria such as *Bifidobacterium*, *Lactobacillus*, *Ruminococcus*, *Faecalibacterium spp.*, *Roseburia,* and *Treponema* are consistently reduced (Cheng, et al., 2020; Huo, et al., 2022).

## Microbes in the prevention and treatment of CRC

The intestinal microbiome plays an integral role in the development and progression of CRC, and manipulation of the microbiome such as the use of probiotics can prevent or treat CRC. The International Scientific Association for Probiotics and Prebiotics panel recommends that the term probiotics be used only in products that have the appropriate number of live microorganisms and that clearly define that the strains can provide reasonably expected benefits to the health of the host (Gibson, et al., 2017). Some commensal microorganisms also have anticancer activity, some of which are considered next-generation probiotics. The mechanisms of action of these beneficial bacteria include regulation of the intestinal microbiota, enhancement of the intestinal epithelial barrier, improvement of intestinal physicochemical conditions, production of beneficial metabolites, and suppression of intestinal inflammation (Molska and Reguła, 2019).

### Bifidobacterium

*Bifidobacterium* is a major member of the gut microbiome in early life, and *Bifidobacteria* belong to the *phylum Actinobacteria*, mainly including *Bifidobacterium infantile*, *Bifidobacterium longata*, and *Bifidobacterium breve* (Saturio, et al., 2021). *Bifidobacterium* metabolizes and produces lactate and acetic acid, which decrease the pH in the gut and thus affect the gut microbiota.

In mice with a point mutation in the adenomatous polyposis coli gene, *Bifidobacterium* colonization reduced polyps by 41% (Liao, et al., 2021). Administration of *Bifidobacterium* CGMCC 15068 attenuated tumorigenesis in a mouse model of inflammation-associated colitis-associated CRC (CAC) (Wang, et al., 2020a). *Bifidobacterium longum* administration caused a decrease in the number of aberrant crypt foci lesions in CRC mice (Fahmy, et al., 2019). *Bifidobacterium* has “protective” anticancer properties comparable with those of cetuximab and trastuzumab and can simultaneously downregulate epidermal growth factor receptor, human epidermal growth factor receptor 2, and prostaglandin-endoperoxide synthase 2, significantly improve the disease activity index, restore colon length, inhibit increased tumor incidence, and prevent tumor progression (Asadollahi, et al., 2020). Bahmani et al. found that cell-free supernatant of *B. bifidum* can inhibit the growth of colon cancer cells (Bahmani, et al., 2019). Consumption of yogurt containing *Bifidobacterium* BB536-year and fructo-oligosaccharides prevented CRC in healthy subjects, and short-chain fatty acids were produced because of increased intake of *Bifidobacterium* BB536-year and fructo-oligosaccharides (Ohara and Suzutani, 2018). The *Bifidobacterium* CGMCC 15068 pretreatment increased the relative abundance of *Akkermansia*, Desulfovibrionaceae, *Romboutsia*, *Turicibacter*, Verrucomicrobiaceae, Ruminococcaceae_UCG_013, Lachnospiraceae_UCG_004, and *Lactobacillus* (Wang, et al., 2020b).

### Lactobacillus

*Lactobacillus* is a common probiotic belonging to facultative anaerobes that are widely found in the human gut and have many beneficial properties, including immunomodulatory, anti-inflammatory, antioxidant, and antiproliferative activities. Some *Lactobacillus* mixtures can inhibit tumor growth (Ghanavati, et al., 2020a; Ghanavati, et al., 2020b). *Lactobacillus rhamnosus GG* (*LGG*) colonization early in life promotes intestinal development, increases tight junction formation, reduces low-grade inflammation, and improves intestinal microbiota composition. In addition, *LGG* colonization regulates the Wingless/Integrated pathway and promotes tumor cell apoptosis, thereby inhibiting tumor formation (Liu, et al., 2022). *LGG* reduces tumor load in a mouse model of intestinal cancer by initiating an anti-tumor immune response (Owens, et al., 2021). *Lactobacillus fermentum* YL-11 exopolysaccharide inhibited the growth of HT-29 cells in tumor-bearing mice (Li, et al., 2022). *Lactobacillus acidophilus* has potential prophylactic effects in a population with a family history of CRC (Zinatizadeh, et al., 2018). *Companilactobacillus crustorum* MN047 can partially inhibit CAC by regulating the intestinal microbiota, reducing inflammation, and enhancing intestinal barrier integrity (Wang, et al., 2021a). *Lactobacillus coryniformis* MXJ32 can inhibit CAC by regulating the intestinal microenvironment and alleviating inflammation and intestinal barrier damage (Wang, et al., 2022).

### Enterococcus faecalis

The role of *Enterococcus faecalis* is controversial. On the one hand, the harmful effect of *E. faecalis* is thought to be mainly related to oxidative stress (Léger, et al., 2019); on the other hand, early colonization of *E. faecalis* in infants contributes to the development of intestinal immunity.

Metabolites produced by the respiration of *E. faecalis* have anti-proliferative activity against the colon cancer cell line HT-29 (Jiao, et al., 2022). *E. faecalis* in the azoxymethane (AOM)/dextran sodium sulfate (DSS) mouse model ameliorates the severity of intestinal inflammation and prevents CAC (Chung, et al., 2019).

### Escherichia coli

*Escherichia coli Nissle* (*EcN*) was isolated in 1917 by Professor Alfred Nissle of Freiburg, Germany, from a young soldier. The soldier did not have infectious diarrhea when he was stationed in southeast Europe, where Shigella was endemic. The strain was designated as *EcN* 1917 (Scaldaferri, et al., 2016).

*EcN* has an important role in apoptosis in colon cancer HT-29 cells through the upregulation of phosphatase and tensin homolog and B-cell lymphoma 2-associated X protein and downregulation of protein kinase B alpha and B-cell lymphoma-extra-large genes (Alizadeh, et al., 2020). Furthermore, *EcN* 1917 is a transformable bacterial vector with probiotic properties for the production and delivery of anticancer agents in microscopic living therapies, including 5-amino acetyl propionate (Chen, et al., 2021a), butyrate (Chiang and Huang, 2021), and the small microcytotoxic protein (Chiang and Hong, 2021).

### Bacteroides fragilis

*Bacteroides fragilis* is a Gram-negative, obligate anaerobic bacterium in which enterotoxigenic *B. fragilis* is considered an oncogenic bacterium, whereas non-virulent *B. fragilis* strains may have probiotic properties.

*B. fragilis* has the potential to prevent inflammatory diseases in the gut. *B. fragilis* plays a protective role in a mouse model of *Clostridium difficile* infection by regulating intestinal microbiota and alleviating barrier disruption, thereby relieving the epithelial stress caused by *C. difficile* and pathogenic colitis (Deng, et al., 2018). Oral treatment with the *B. fragilis* ZY-312 strain improves the symptoms of antibiotic-associated diarrhea by increasing the abundance of a specific symbiotic microbiota. These changes were consistent with the restoration of intestinal barrier function and enterocyte regeneration in antibiotic-associated diarrhea rats. In addition, polysaccharide A in *B. fragilis* ameliorates abnormal voriconazole metabolism by inhibiting toll-like receptor 4-mediated nuclear factor kappa-B (NF-κB) transcription and regulating the expression of drug metabolism enzymes and transporters, which therefore can be used for the clinical adjuvant treatment (Wang, et al., 2021b).

### Streptococcus thermophilus

*Streptococcus thermophilus* is a Gram-positive bacterium that is widely used as a starter in the dairy industry as well as in many traditionally fermented products.

Transoral gavage of *S. thermophilus* significantly reduced tumor formation in mice with a point mutation in the adenomatous polyposis coli gene and mice injected with AOM. The proliferation of CRC cells was inhibited when cocultured with *S. thermophilus* or their conditioned medium. β-Galactosidase is a key protein produced by *S. thermophilus*. It inhibits cell proliferation, reduces colony formation, induces cell cycle arrest, promotes apoptosis of CRC cells, and delays the growth of CRC xenografts while increasing the intestinal abundance of probiotics, including *Bifidobacterium* and *Lactobacillus* (Li, et al., 2021a). Two *S. thermophilus* strains, M17PTZA496 and TH982, have *in vitro* probiotic properties as well as anticancer activity, simultaneously producing folate and inhibiting in HT-29 cells (Tarrah, et al., 2018).

### Clostridium butyricum

The *Clostridium butyricum* (*CB*) cell-free supernatant and Bacillus subtilis inhibited the development of dimethylhydrazine-induced CRC *in vivo*. *CB* inhibits the progression of CRC, improves inflammation in AOM/DSS mice, changes intestinal microbiota composition, and regulates the expression of MyD88 and NF-κB (Zhou, et al., 2022). *CB* reduces *Firmicutes*/*Bacteroidetes* ratio, increases the relative abundance of probiotics, reduces colitis, reduces CRC incidence and tumor size, increases apoptosis of tumor cells, reduces cytokines including tumor necrosis factor-alpha (TNF-α) and interleukin (IL)-6, reduces cyclooxygenase-2, reduces phosphorylation of NF-κB, reduces B-cell lymphoma two protein, and increases B-cell lymphoma 2-associated X protein expression (Liu, et al., 2020). *CB* inhibits enterotoxigenic *B. fragilis* growth in planktonic culture and exhibits anti-biofilm effects by inhibiting biofilm development, breaking down preformed biofilms, and reducing the metabolic activity of cells in biofilms and therefore can serve as a biotherapeutic agent (Shin, et al., 2020).

### Faecalibacterium prausnitzii

*Faecalibacterium prausnitzii* significantly reduced the frequency and formation of abnormal colonic crypt foci in rat AOM-induced CRC. Furthermore, the application of *F. prausnitzii* reduced the level of lipid peroxidation in colonic tissues. Cell-free supernatant of *F. prausnitzii* inhibited HCT116 cell growth in a dose-dependent manner. Meanwhile, *F. prausnitzii* regulated the rat gut microbiota and increased diversity (Dikeocha, et al., 2022).

## Microbes and the tumor microenvironment

CRC is a tumor infiltrated by effector memory lymphocytes, and the TME is the key to cancer immunotherapy. The components of TME in CRC include tumor cells, blood vessels, extracellular matrix, fibroblasts, lymphocytes, bone marrow-derived suppressor cells, and signaling molecules (Chen, et al., 2021b). Some bacteria proliferate in the TME and alter it to promote tumor progression (Kasper, et al., 2020); accordingly, some bacteria contribute to improving the TME and thus exert anti-cancer activity. Currently, immunotherapies for CRC such as programmed cell death protein 1 (PD-1) or cytotoxic T lymphocyte-associated antigen-4 (CTLA-4) blockers act mainly through T cells (Makaremi, et al., 2021), while beneficial bacteria act synergistically with immune checkpoint blockade (ICB) by activating immune cells and regulating cytokine secretion.

### Immunocytes

**FIGURE 1:**
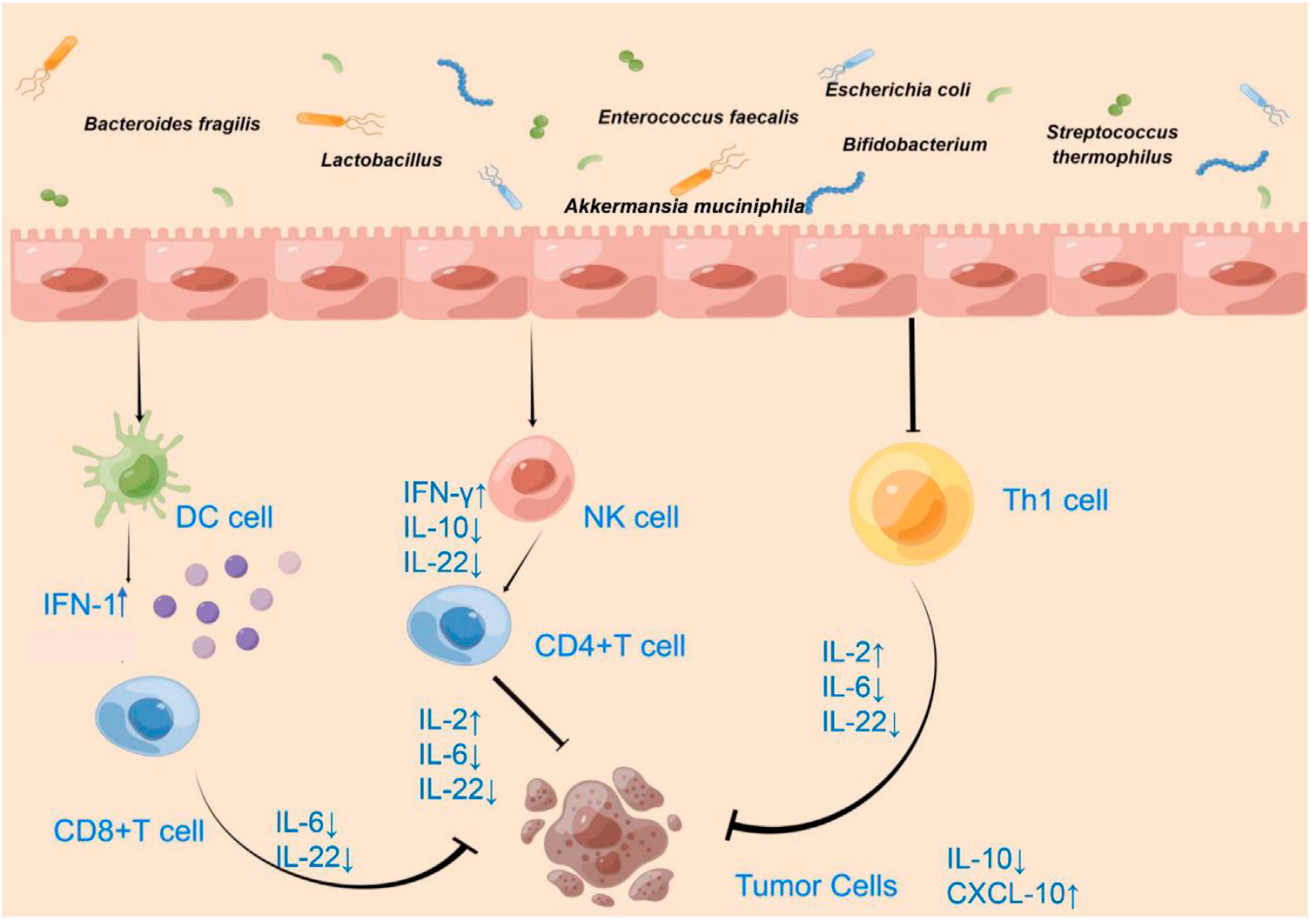
Effect of microbiota on the immune microenvironment of CRC.

Immune cell analysis of spleen and tumor tissues showed that short *Bifidobacterium* strains alone enhanced antitumor immunity by the increasing cluster of differentiation (CD)8+ T-cell and effector CD8^+^ T-cell numbers and by increasing CD8^+^ regulatory T cells (Treg) and effector CD8+/Treg ratios (Table 1) (Yoon, et al., 2021). Some *Bifidobacterium spp.* cause the reduction of proinflammatory factor IL-6 (Table 1) (Figure 1) and the high accumulation of mature DCs, helper T cells (Th), and cytotoxic T cells (CTLs) at tumor sites under IL-6-deficient conditions (Table 1) (Singh, et al., 2020; Chen, et al., 2021c; Cui, et al., 2022). IL-6 promotes metastatic colonization of CRC cells by modulating the tumor immune microenvironment, and in primary tumors, CRC patients with low IL-6 expression exhibit prolonged disease-free survival (Toyoshima, et al., 2019). In addition, the *Bifidobacterium* strain *Bifidobacterium breve* JCM92 regulates the recruitment of immune cells in the TME to increase antitumor immunity, enhancing the antitumor effect of oxaliplatin (Yoon, et al., 2021).

**TABLE 1:** Influence of intestinal microorganisms on the immune microenvironment of colorectal tumors.

Microbes	Immunocytes	Cytokines	References
*Bifidobacterium*	CD8^+^ T-cell	IL-6	Yoon, et al. (2021); Singh, et al. (2020); Chen, et al. (2021); Cui, et al. (2022)
*Lactobacillus*	CD8^+^ T-cell, DC	IFN-γ, IL-10, IL-22, CXCL9, CXCL10, CXCL11, CCL20	Owens, et al. (2021); Zhuo, et al. (2019); Zhang, et al. (2022); Shang, et al. (2020); Kawanabe-Matsuda, et al. (2022); Jacouton et al. (2017); Li, et al. (2021a); Si, et al. (2022)
*E. faecalis*	T cell		Griffin, et al. (2021)
*E. coli*	Th1 cell, CTL, Treg, macrophage, type 1 innate lymphocyte		Shi, et al. (2019)
*AKK*	CTL, Treg, macrophage	IFN-γ, IL-2	Shi, et al. (2020b); Fan, et al. (2021); Wang, et al. (2020a)

Increased colonic CD8^+^ T-cell responses were detected in *LGG*-supplemented mice, induction of CD8^+^ T cells was dependent on toll-like receptor 2-mediated activation, and *LGG* reduced tumor burden in a mouse intestinal cancer model in a CD8^+^ T cell-dependent manner (Table 1) (Owens, et al., 2021). Meanwhile, *L. acidophilus* lysates had immunomodulatory effects by inhibiting the IL-10 expression levels in M2 polarization and Lipopolysaccharide-activated Raw264.7 macrophages (Table 1) (Zhuo, et al., 2019). *L. paracasei* sh2020–triggered antitumor immune response is dependent on CD8^+^ T cells. *In vitro* and *in vivo* studies have shown that the *L. paracasei* sh2020 enhanced CD8^+^ T-cell recruitment, with increased T-cell infiltration, and improved the poorly infiltrated TME, thus promoting immunotherapy (Table 1) (Zhang, et al., 2022). A probiotic mixture containing *B. longum*, *B. bifidum*, *L. acidophilus*, *L. plantarum,* and other components inhibits the invasion, migration, and proliferation of CT26 cells and may exert anti-tumor effects by inducing CD8^+^ T cell immune responses (Table 1) (Shang, et al., 2020).

*E. faecalis* CECT7121 can stimulate local mucosal immunity and adhere to intestinal epithelial cells, which can stimulate the mucosal immune system and increase the number of IgA + cells in the lamina propria, without inducing an epithelial inflammatory response (Castro et al., 2016). Immunoreactive muropeptides are prevalent throughout human-associated enterococcal species. *Enterococci* with unique NlpC/p60 peptidoglycan hydrolase activity produce nucleotide-binding oligomerization domain two active muropeptides and modulate the effects of ICB immunotherapy *in vivo*, and tumors treated with active muropeptide-L, D isoforms show a significant increase in the proportion of intra-tumor T cells (Table 1) (Griffin, et al., 2021). Pretreatment with *E. faecalis* significantly attenuated nucleotide-binding oligomerization domain-like receptor protein 3 (NLRP3) inflammasome activation in macrophages induced by intestinal commensal microorganisms and attenuated colitis and CAC (Chung, et al., 2019).

Oral administration of *EcN* alone inhibited tumor growth, which may be due to increased tumor-specific effector T-cell infiltration and improved tumor immunosuppression. Combination treatment of transforming growth factor-β blocker with *EcN* appeared to restore tumor-infiltrating CTL-disrupted cancer cells and increased the proportion of tumor-infiltrating CD3^+^CD8+IFN-γ+CTL (Table 1) (Shi, et al., 2019). *EcN* 541–15 implantation affected the TME, with a decrease in myeloid infiltration, including tumor-associated macrophages, mononuclear myeloid-derived suppressor cells, and polymorphonuclear myeloid-derived suppressor cells, and a decrease in Tregs in tumors of *EcN* 541–15 implanted mice. Changes in lymphocyte recruitment to tumors were found in 541–15 colonized mice, with an increase in Th1 cells, CTLs, and type 1 innate lymphocytes (Table 1) (Zegarra Ruiz, et al., 2022). Furthermore, *B. fragilis* can alter the Treg/Th-17 balance by counteracting the lipopolysaccharide-induced inflammatory responses, thereby exerting immunomodulatory effects (Chang, et al., 2017).

Oral *Akkermansia muciniphila* (*AKK*) triggers antitumor immune responses, induces tumor shrinkage, and prolongs median survival in tumor-bearing mice, and While *AKK* treatment is successful in lowering the proportion of Treg cells in the TME, *AKK* alone attracts a higher proportion of CTL than the preadministration of IL-2 combined with *AKK*. In addition to significantly lowering the proportion of CD133+ cells in tumor tissue and diminishing tumor stem cell-like potency, the combination of IL-2 and *AKK* is more effective than monotherapy at controlling Treg levels (Table 1) (Shi, et al., 2020a). Supplementation with *AKK* inhibits colon tumorigenesis in mice with a point mutation in the adenomatous polyposis coli gene and the growth of implanted HCT116 or CT26 tumors in nude mice. *AKK* promotes the enrichment of M1-like macrophages *in vivo* and *in vitro* in an NLRP3-dependent manner (Table 1) (Fan, et al., 2021). A protein extracted from *AKK* named Amuc_1100 significantly increased the percentage of CTL in mesenteric lymph nodes and the colon, thereby exacerbating apoptosis in tumor cells (Table 1) (Wang, et al., 2020a).

### Cytokines

*Lactobacillus paracasei* sh2020 promotes the expression of T cell chemokines such as chemokine (C-X-C motif) ligand (CXCL)9, CXCL10, and CXCL11. In an *in vitro* tumor cell culture model, stimulation of *L. paracasei* sh2020 may lead to increased production of CXCL10, a T helper type 1 chemokine that controls the entry of major anti-tumor immune cells into tumor bed (Table 1) (Figure 1) (Zhang, et al., 2022). *Lactobacillus*-derived exopolysaccharide-R1-induced T cells can infiltrate CCL20-producing tumors and produce IFN-γ, enhancing the effect of ICB therapy in producing CCL20 tumors in mice (Table 1) (Figure 1) (Kawanabe-Matsuda, et al., 2022). *L. casei* BL23 mediated immunomodulatory effects through the downregulation of IL-22 cytokines (Table 1) (Figure 1) (Jacouton et al., 2017). In the *in situ* ligated intestine loop model, stimulation of *B. breve* triggered upregulation of DC-associated chemokine CCL20 expression and increased DCs recruitment in the intestinal villi, enhancing DC-derived IL-12 secretion on the antitumor effect of *B. breve* (Figure 1) (Li, et al., 2021b).

In the AOM/DSS-induced CAC mouse model, *B. fragilis* acted in a polysaccharide A-dependent manner, with a significant reduction in the number and size of tumors in the colon of the *B. fragilis*-treated mice, and reduced expression of chemokine receptor (CCR) five was observed in the colon tissue of *B. fragilis*-treated mice (Lee, et al., 2018).

Combined administration of *AKK* and IL-2 has a better tumor suppressive effect than single administration, altering the TME, except affecting immune cells, and inducing the production of pro-inflammatory cytokines, significantly increasing IFN-γ and IL-2 levels in tumor tissues (Table 1) (Figure 1) (Shi, et al., 2020b).

### Combination of gut microbes and immune checkpoint inhibitors

*Bifidobacteria* promote local anti-CD47 to tumor immunotherapy by accumulating in the TME, which effectively stimulates the stimulator of interferon genes signaling and increases the cross-initiation of dendritic cells (DCs) after anti-CD47 treatment. Type I interferon (IFN) signaling in DCs is critical for the therapeutic outcome of *Bifidobacterium*-promoted CD47 blockade. After CD47 blockade, type I IFN can be upregulated in bone marrow-derived DCs co-cultured with tumor cells and *Bifidobacterium* (Figure 1) (Shi, et al., 2020a). Intratumor cytokine expression showed that mice treated with PD-1 blocker and *B. breve* JCM92 had higher IFN-γ and IL-2 expression compared to mice treated with PD-1 blocker alone (Figure 1) (Yoon, et al., 2021).

In addition, antigenic mimicry of gut microbes affects T-cell immunity and contributes to cross-reactive antitumor responses. T cells targeting an epitope called SVYRYYGL expressed in the commensal bacterium *B. breve* cross-react with the model neoantigen SIYRYYGL. In a mouse melanoma model, SVYRYYGL-reactive T cells were reduced in mice lacking *B. breve*, and SVYRYYGL -specific T cells recognized *in vivo* melanomas expressing SIYRYYGL (Bessell, et al., 2020). *Bifidobacterium pseudolongum* isolated from ICB-treated CRC tumors promotes conventional DCs-dependent Th1 cell circuits, thereby greatly enhancing the effects of ICB therapy in mouse models of intestinal and epithelial tumors, and modulates the enhanced immunotherapeutic response through the production of the metabolite inosine (Mager, et al., 2020).

Oral live *LGG* enhances the antitumor activity of anti-PD-1 immunotherapy by increasing tumor-infiltrating DCs and T cells. In addition, *LGG* combined with PD-1 treatment shifted the gut microbial community towards *Lactobacillus murinus and Bacteroides uniformis* enrichment, increasing DCs activation and CD8^+^ tumor recruitment (Table 1) (Si, et al., 2022). Dietary intake of *Lactobacillus*-derived exopolysaccharide induces CCR6+CD8^+^ T cells in the Peyer’s patches, enhancing the antitumor effect of anti-CTLA-4 or anti-PD-1 monoclonal antibodies on CCL20-expressing tumors (Kawanabe-Matsuda, et al., 2022). *L. acidophilus* lysates enhanced the antitumor activity of CTLA-4 monoclonal antibodies in a mouse model. In the TME, CD8^+^ T cells were increased, effector memory T cells (CD44^+^CD8^+^CD62L+) were increased, and Treg (CD4^+^CD25+FoxP3+) and M2 macrophages (F4/80+CD206+) were decreased (Table 1) (Zhuo, et al., 2019). In mice and patients, the T-cell response of *Bacteroides thetaiotaomicron* or *B. fragilis* was correlated with the efficacy of CTLA-4 blockade. Tumors from antibiotic-treated mice or germ-free mice did not respond to CTLA-4 blockade. This absence of response can be improved by the use of *B. fragilis* for gavage, polysaccharide application, or adoptive transfer of *B. fragilis*. Fecal microbial transplantation from humans to mice confirmed that the treatment of melanoma patients with antibodies directed against CTLA-4 favored the growth of *B. fragilis* with anticancer properties (Vétizou, et al., 2015).

## Conclusion

The intestinal microbiota is closely associated with the development and progression of CRC, and the prevention and treatment of CRC can be facilitated by the intake of specific intestinal bacteria, including probiotics. These bacteria promote apoptosis, inhibit tumor cell proliferation, and play an important role in the TME, either by themselves or by producing beneficial metabolites. In addition to tumor cells, the TME also includes immune cells, and intestinal microorganisms are also members of the microenvironment. The TME is closely related to the development and progression of tumors.

We review some beneficial bacteria that act on DCs, NK cells, cytotoxic T cells, and helper T cells to promote the secretion of the pro-tumor cytokines IFN-1, IFN-γ, and IL-2 and down-regulate the secretion of TNF-α, IL-6, IL-10, and IL-22, thus exerting anti-tumor immune effects. Targeting and manipulating the cells and factors in the TME can actively treat tumors. Focusing on the active role of the gut microbiota in the TME and combining the gut microbiota with immunotherapy is conducive to improving current immunosuppressive therapies, demonstrating their promise, and facilitating the progress of tumor immunotherapy with the use of the gut microbiota in clinical practice. Meanwhile, microbiota associated with the TME is bound to be further developed, however, the positive effects of these gut microbes on the TME and the therapeutic effects of beneficial combinations of gut microbes and immunosuppressive agents on tumors need to be demonstrated in more animal and clinical experiments.

## Abbreviations

AKK, *Akkermansia muciniphila*; AOM, Azoxymethane; CAC, Colitis-associated CRC; *CB*, *Clostridium butyricum;* CCR, Chemokine receptor; CD, Cluster of differentiation of differentiation; CRC, Colorectal cancer; CTLA-4, Cytotoxic T lymphocyte-associated antigen 4; CXCL, Chemokine (C-X-C motif) ligand; DC, Dendritic cell; DSS, Dextran sodium sulfate; EcN, *Escherichia coli Nissle;* IFN, Interferon; IL, Interleukin; LGG, *Lactobacillus rhamnosus GG;*NF-κB, Nuclear factor kappa-B; NLRP3, Nucleotide-binding oligomerization domain-like receptor protein 3; PD-1, Programmed cell death protein 1; Th, Helper T cell; TME, Tumor microenvironment; TNF-α, Tumor necrosis factor-alpha; Treg, Regulatory T cell.
